# Construction and validation of a rat model of acute necrotizing pancreatitis-associated intestinal injury

**DOI:** 10.1152/ajpgi.00262.2023

**Published:** 2024-05-14

**Authors:** Haojie Jiang, Weidong Xie, Qinbo Chen, Yiling Li, Zhen Yu, Naxin Liu

**Affiliations:** ^1^Wenzhou Medical University, Wenzhou, People’s Republic of China; ^2^Department of Gastrointestinal Surgery, The First Affiliated Hospital of Wenzhou Medical University, Wenzhou, People’s Republic of China; ^3^Zhejiang Key Laboratory of Intelligent Cancer Biomarker Discovery and Translation, First Affiliated Hospital, Wenzhou Medical University, Wenzhou, People’s Republic of China; ^4^Shanghai Tenth People’s Hospital, Shanghai, People’s Republic of China

**Keywords:** acute necrotizing pancreatitis (ANP), intestinal injury, rat model, pancreatitis-associated ascites fluid (PAAF), HIPPO signaling pathway

## Abstract

Acute pancreatitis (AP) is an acute inflammatory reaction of the pancreatic tissue, which involves auto-digestion, edema, hemorrhage, and necrosis. AP can be categorized into mild, moderately severe, and severe AP, with severe pancreatitis also referred to as acute necrotizing pancreatitis (ANP). ANP is characterized by the accumulation of necrotic material in the peritoneal cavity. This can result in intestinal injury. However, the mechanism of ANP-associated intestinal injury remains unclear. We established an ANP-associated intestinal injury rat model (ANP-IR model) by injecting pancreatitis-associated ascites fluid (PAAF) and necrotic pancreatic tissue at various proportions into the triangular area formed by the left renal artery and ureter. The feasibility of the ANP-IR model was verified by comparing the similar changes in indicators of intestinal inflammation and barrier function between the two rat models. In addition, we detected changes in apoptosis levels and YAP protein expression in the ileal tissues of rats in each group and validated them in vitro in rat epithelial crypt cells (IEC-6) to further explore the potential injury mechanisms of ANP-associated intestinal injury. We also collected clinical data from patients with ANP to validate the effects of PAAF and pancreatic necrosis on intestinal injury. Our findings offer a theoretical basis for restricting the buildup of peritoneal necrosis in individuals with ANP, thus promoting the restoration of intestinal function and enhancing treatment efficacy. The use of the ANP-IR model in further studies can help us better understand the mechanism and treatment of ANP-associated intestinal injury.

**NEW & NOTEWORTHY** We constructed a rat model of acute necrotizing pancreatitis-associated intestinal injury and verified its feasibility. In addition, we identified the mechanism by which necrotic pancreatic tissue and pancreatitis-associated ascites fluid (PAAF) cause intestinal injury through the HIPPO signaling pathway.

## Graphical abstract

**Figure:**
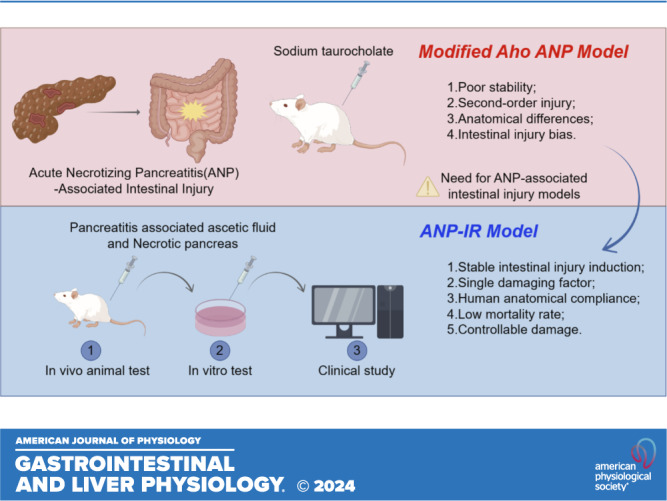

## INTRODUCTION

Acute pancreatitis (AP) is an inflammatory condition affecting the pancreatic tissue and characterized by autodigestion, edema, bleeding, and even necrosis. Depending on the degree of severity, AP can be classified into three categories: mild, moderately severe, and severe; severe acute pancreatitis (SAP) is also referred to as acute necrotizing pancreatitis (ANP) (1). With the disease progression, systemic or local complications occur, resulting in two mortality peaks. The first peak is associated with multiple organ failure syndrome (MODS) and occurs in the early stages of systemic inflammatory response syndrome, whereas the second peak is associated with infection-associated MODS (2–5). High mortality in the late stages of AP is often attributed to severe infection, with pathogenic bacteria typically originating from the intestines (6). Imbalances in intestinal flora are recognized as a primary factor contributing to intestinal injury. Intestinal barrier dysfunction and inflammatory damage play pivotal roles in the development and dissemination of AP-associated complications, reinforcing the central hypothesis that the gut is a critical site for infection (7–9). Therefore, elucidating the mechanisms of intestinal injury associated with ANP is crucial.

In the study of AP, the use of modified Aho rat models enables the simulation of clinical biliary reflux pancreatitis (10). However, the modified Aho rat model is not designed specifically to study ANP-associated intestinal injury. Furthermore, the model does not consistently induce intestinal injury, which would be a waste of resources during the study. In addition, this technique is complex and associated with a high mortality rate, often failing to simulate the second peak of ANP mortality. In this study, an ANP-associated intestinal injury model that simulated intestinal injury resulting from acute fluid accumulation and necrotic material buildup was designed by injecting a mixture of pancreatitis-associated ascites fluid (PAAF) and necrotic pancreatic tissue in varying proportions into the triangular area formed by the left renal artery and ureter in rats. Liu et al. (11) investigated the effect of peritoneal macrophage polarization on peritoneal puncture drainage for ANP by adding peritoneal lavage fluid to the culture medium to simulate the inflammatory environment of the peritoneum (11, 12). In the present study, we also tried to add the above-mentioned necrotic pancreatic tissues and PAAF directly to the culture medium to simulate acute fluid accumulation and necrotic accumulation in vitro.

The Hippo signaling pathway is a pivotal cellular signaling cascade that regulates the expression of target genes to govern various cellular processes. This pathway comprises several core components, including fruit fly Hippo and Warts kinases or human mammalian sterile-20-like 1/2 (MST1/2) and large tumor suppressor 1/2 (LATS1/2) kinases. In addition, this pathway involves yes-associated protein (YAP) and TAZ protein as effector components (13). The tight junction protein zonula occludens 1 (ZO-1) plays a crucial role in the formation and maintenance of tight intercellular connections. ZO-1 interacts with the Hippo signaling pathway to regulate cell proliferation, epithelial-mesenchymal transformation, and apoptosis (14–17). Thus, the Hippo signaling pathway and the closely related protein ZO-1 can be investigated as potential targets for understanding the mechanisms underlying ANP-associated intestinal injury.

## MATERIALS AND METHODS

### Reagents

All chemicals and reagents were of analytical grade. Anti-glyceraldehyde-3-phosohate dehydrogenase (GAPDH) (GB15002) was obtained from Affinity. Anti-YAP (D8H1X, 14074 T), caspase-3 (Cat. No. 9662S), cleaved-caspase-3 (Asp175, Cat. No. 9661S), Bax (Cat. No. 41162), and Bcl-2 (Cat. No. 4223) were all obtained from Cell Signaling Technology (CST, MA). Anti-aquaporin-1 (AQP-1, A4195) and anti-ZO-1 (A11417) were obtained from ABclonal.

### Animal Models

The experimental protocol was approved by the Institute Animal Care and Use Committee of Wenzhou Medical University (WYYY-AEC-2023-108), and all experimental procedures described were performed in accordance with established institutional guidelines and approved protocols. Rats were housed in a cage (four rats per cage) in a temperature-controlled room with a 12-h light-dark cycle. Food and water were available ad libitum.

Forty male Sprague–Dawley rats were obtained from the Animal Laboratory Center of the First Affiliated Hospital of Wenzhou Medical University (Wenzhou, CN). The rats were starved for 24 h before surgery but were allowed to drink water freely during this period. They were then injected with 2% pentobarbital (0.3 mL/100 g, subcutaneously) in the abdomen and restrained in the supine position.

**Figure 1.**
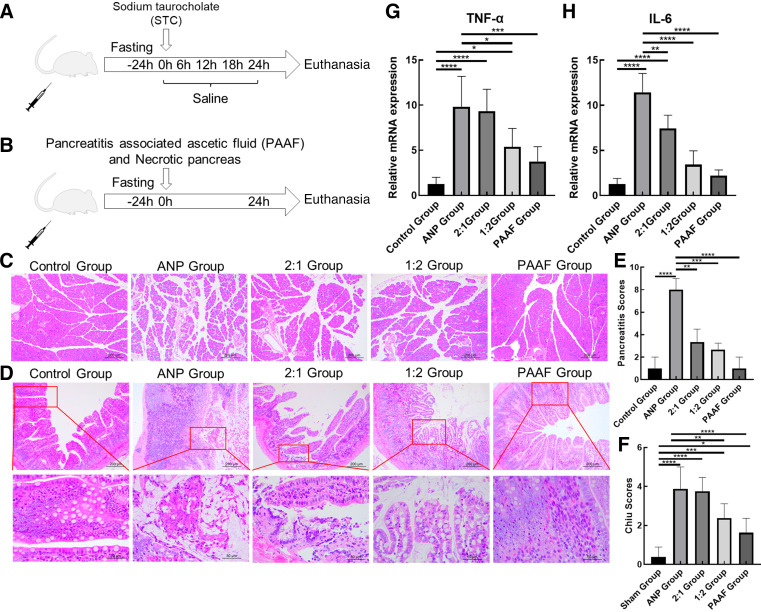
Activity levels decreased and intestinal inflammatory responses increased in different groups*. A* and *B:* schematics of animal grouping and experimental protocols. *C*: histological alterations observed in the pancreas (H&E staining, scale bar = 200 μm). *D:* histological alterations observed in the mucosal lining of the small intestine (H&E staining, scale bar = 200 μm and 50 μm). *E*: the pancreas injury score was graded using Schmidt scores. *F*: the intestinal mucosal injury was graded using Chiu’s scores. *G* and *H*: RT-qPCR results indicating the expression of TNF-α and IL-6. H&E, hematoxylin-eosin. Data are expressed as the means ± SD, *n* = 8 in each group, *P < 0.05, **P < 0.01, ***P < 0.001, ****P < 0.0001.

The ANP rat model was established using a modified Aho method, involving the injection of 3.5% sodium taurocholate (86339 Sigma, America) into the bile duct at a constant rate (Fig. 1*A*).

For the acute necrotizing pancreatitis-associated intestinal injury model (ANP-IR model) group, we injected necrotic pancreas and PAAF, which were collected from the ANP group, into the triangular region formed by the left renal artery and ureter at a constant rate. The injection volume used was 0.1 mL/100 g (Fig. 1*B*). Supplemental Fig. S1 presents photographs taken during the fabrication of the ANP-IR model.

### Grouping

During the initial phase of the study, we selected various mixtures with different ratios and found that the difference in intestinal injury was most significant between the ANP-IR models constructed by injecting PAAF alone, 1:2 and 2:1 mixtures. Therefore, the rats were randomly divided into the following five groups (*n* = 8 in each group): *1*) control group; *2*) modified Aho method ANP group (ANP group); *3*) necrotic pancreas and PAAF 2:1 mixture group (2:1 group); *4*) necrotic pancreas and PAAF 1:2 mixture group (1:2 group); and *5)* PAAF group.

### Histological Analysis (Morphological Analysis)

Tissue sections of the pancreas and intestine were stained with hematoxylin and eosin and observed under a light-emitting diode (LED) microscope system. Schmidt et al. (18) describe a pancreatic pathological grading system that considers various factors, such as pancreatic edema, acinar necrosis, hemorrhage and fat necrosis, inflammation, and perivascular infiltration, which is used to evaluate pancreas injury. The Chiu score is used to evaluate mucosal injury (19). The scoring system includes normal mucosa, 0; subepithelial space at villus tip, 1; widening of the subepithelial space with moderate lifting of the epithelial layer, 2; massive epithelial lifting down-villus slides and ulceration at villus tips, 3; denuded villi with dilated capillaries and increased cellularity of lamina propria, 4; and digestion and disintegration of lamina propria in the villi with hemorrhage and ulceration, 5.

### Measurement of Serum d-Lactate and Diamine Oxidase Levels

The concentrations of DAO and d-Lac in the serum were detected using DAO (BP-E31664, BOYUN Biotech) and d-Lac (Cat. No. H263, NJJCBIO) enzyme-linked immunosorbent assay (ELISA) assay kits according to the manufacturer’s instructions.

### Immunohistochemistry

Immunohistochemistry was performed to examine the expression of ZO-1 and AQP-1 in the intestinal tissue. Xylene was used for deparaffinizing paraffin-embedded sections (4 μm), which were then rehydrated in descending alcohol solutions. Antigen retrieval was performed by incubating the sections in sodium citrate (0.01 mol/L, pH 6.0) at 100°C for 15 min. To eliminate the effects of endogenous peroxidases, hydrogen peroxide (3%) (AR1108, BOSTER) was used. After blocking, the sections were incubated with the anti-ZO-1 antibody or anti-AQP-1 antibody overnight at 4°C. We washed the sections and incubated them at 37°C for 1 h with secondary antibodies. The immunocomplexes were visualized with diaminobenzidine (ZLI-9017, ZSGB BIO) as brown pigments. Slides were counterstained with hematoxylin, dehydrated in alcohol, and cleared in xylene. Light microscopy was used to capture representative images.

### Western Blot

For total protein preparation, the animal tissues and cells were lysed with RIPA lysis buffer supplemented with PMSF (GK10023, GLPBIO) on ice. Then, subjected to centrifugation to obtain supernatants. Protein samples were denatured in a loading buffer and separated using SDS-PAGE. Subsequently, the proteins were electroblotted onto PVDF membranes (Millipore, Billerica). The membranes were then incubated with 5% defatted milk and further incubated with various primary antibodies at 4°C overnight. Subsequently, the proteins were incubated for another 1 h with peroxidase-conjugated secondary antibodies under ambient temperature. The target proteins were detected using the enhanced chemiluminescence detection kit, and ImageJ software was used for analyses. GAPDH was used as an internal control.

### Real-Time Polymerase Chain Reaction

To 100 mg of the rat ileum tissue, 1 mL of TRIzol was added. Then, the tissue was thoroughly grounded using a high-throughput tissue grinder, followed by the addition of 100 μL of chloroform. The mixture was allowed to stand at room temperature for 10 min and then centrifuged at 12,000 rpm at 4°C for 20 min. The supernatant was collected and mixed with an equal amount of isopropanol. Then, the mixture was incubated at room temperature for 10 min and centrifuged for 15 min. After discarding the supernatant, 1 mL of anhydrous ethanol was added, and the mixture was stirred and then centrifuged for 5 min. The supernatant was discarded and air-dried. Then, we added 20 μL of DEPC water (BL510B, Biosharp), detected the quality and concentration of the extracted total RNA by using a spectrophotometer, and stored the mixture at −80°C for further use. Reverse transcription was performed in accordance with the manufacturer’s instructions (R333, Vazyme), and reverse transcription products were stored at −20°C. The qPCR kit (Q712, Vazyme) was used, and three replicate wells were set up for each sample to obtain the mean Ct value. The 2^−ΔΔCt^ method was used to calculate the relative mRNA expression level. The primer sequences are provided in Supplemental Table S1.

### Cell Culture Conditions

Rat small intestinal crypt epithelial cells (IEC-6) were obtained from iCell Bioscience (iCell-r016). The IEC-6 cells were maintained in DMEM supplemented with 10% fetal bovine serum (10270-106, Gibco), 10 μg/mL insulin (BS901, BIOSHARP), and 100 U/mL penicillin-streptomycin (15140122, Gibco). When the cells reached 80%–90% confluence, they were digested and subcultured for the subsequent treatments.

### Cell Viability Analysis

Cell viability was assessed using the cell counting kit-8 (GK10001, GLPBIO). IEC-6 cells were seeded in 96-well plates at a density of 10^4^ cells/well. Subsequently, the cells were treated with a mixture of PAAF and necrotic pancreatic tissues in different proportions for 24 h. The premixed solution containing 10% CCK-8 and fresh culture medium was added, and the cells were incubated at 37°C for 2 h. Absorbance at 450 nm was measured using a microplate reader.

### Plasmid Transfection

For transfection, the cells were seeded in a six-well plate at a density of 1 × 10^5^ cells per well in a complete growth medium. When the cells reached 70%–80% confluence, they were washed with phosphate-buffered saline (PBS), and the medium was replaced with serum-free DMEM. Plasmid DNA was then mixed with polyethyleneimine (PEI) in a 1:2 ratio. The mixture was then incubated at room temperature for 20 min to allow complex formation.

After incubation, the DNA–transfection reagent complex was added dropwise to each well containing cells in DMEM. The plate was gently vortexed to ensure uniform distribution and then incubated at 37°C in a humidified atmosphere with 5% CO_2_ for 48 h. Then, the medium was removed and the cells were washed with PBS to remove remaining transfection complexes. Furthermore, 1 mL of medium and 2 μL of LG418 were added to each well. The solution was changed, and 2 μL of LG418 was added daily until all normal cells in the control wells were dead. The remaining transfected cells were cultured in DMEM medium supplemented with 10% fetal bovine serum.

### Annexin V/Propidium Iodide Staining for Apoptosis

Cell apoptosis was determined using the Annexin V-FITC/propidium iodide (PI) apoptosis detection kit (FXP018; 4 A BIOTECH). Briefly, the cells were harvested and washed twice with ice-cold PBS after treatment. Then, the solution was resuspended in a binding buffer, and 100 μL of the solution was mixed with 5 μL Annexin V/FITC and incubated at room temperature in the dark for 5 min. Finally, 10 μL of PI and 400 μL of PBS were added for immediate flow detection. The samples were analyzed within 1 h using a flow cytometer (Beckman Coulter).

### Patients

Patients diagnosed with acute pancreatitis at the Pancreatitis Diagnosis and Treatment Center of the First Affiliated Hospital of Wenzhou Medical University were included in this study from 2017 to 2020. The inclusion criteria included patients who *1*) had modified Balthazar CT score of at least 4; *2*) did not exceed 72 h from onset of symptoms to hospital admission; and *3*) had complete and accurate personal and clinical information. We excluded patients who *1*) had a history of intestinal surgery; *2*) had intestinal injuries caused by other factors; *3*) had ascites caused by other reasons, such as liver cirrhosis; *4*) were pregnant or breastfeeding; and *5*) were suffering from other serious diseases (e.g., malignant tumors, severe heart disease, etc.) (Supplemental Fig. S2). The clinical study was approved by the Ethics Committee in Clinical Research (ECCR) of the First Affiliated Hospital of Wenzhou Medical University (KY2024-R048).

### Modified Balthazar CT Score

Modified Balthazar CT score evaluates not only the pancreatic lesions but also various factors, including peripancreatic fat inflammation, pancreatic effusion, and granulomatous inflammation. This comprehensive approach to assessment can more accurately predict the overall prognosis of patients. It comprises three parts: the first part (Part 1) involves grading of AP: normal pancreas, 0; Pancreatic and/or peripancreatic inflammatory changes, 2; Single or multiple areas of fluid collection or peripancreatic fat necrosis, 4. The second part (Part 2) involves the determination of the degree of pancreatic necrosis: No necrosis, 0; Extent of necrosis ≤ 30%, 2; Extent of necrosis ≥ 30%, 4. The third part (Part 3) involves extrapancreatic complications: no extrapancreatic complications, 0; Pleural effusion, ascites, gastric outflow obstruction, pseudocyst bleeding, splenic vein or portal vein thrombosis, 2.

The sum of the three parts was the modified Balthazar CT score and the score ≥4 was considered as moderately severe acute pancreatitis (MSAP) or SAP.

### Statistical Analysis

Data are presented as the mean ± standard deviation (SD) of three independent experiments. One-way ANOVA was applied to detect significant differences between experimental groups, followed by Tukey’s post hoc test. All statistical analyses were conducted using GraphPad Prism 8.0 (GraphPad Software, CA), with *P* values less than 0.05 considered to be statistically significant.

## RESULTS

### Activity Levels Decreased and Intestinal Inflammatory Response Increased in Different Groups

Regarding activity levels, the control group exhibited no postoperative changes. However, the other four groups displayed varying degrees of decrease in activity, which was manifested by reduced exercise, rapid and shallow respiration, and even coma. The ANP group and the 2:1 group exhibited the most pronounced reduction in activity levels.

At 24 h after operation, the survival rate of rats in ANP group was 61.5%. In the 2:1 group, it was 80%, and in the remaining three groups, all rats survived. We observed pancreatic hemorrhage, edema, and substantial necrosis in rats in the ANP group. However, no significant necrosis was noted in the pancreas of rats in the other groups (Fig. 1, *C* and *E*). In addition, the ileum of rats in the ANP group displayed edema, with no or little content in the intestinal lumen. Furthermore, the ileum of rats in the ANP-IR group displayed different degrees of edema, with the ileal status of rats in the 2:1 group being similar to that of the ANP group and rats in the PAAF group being similar to those in the control group. The pancreas and ileal condition of rats in the control group exhibited no differences before and after surgery.

The ileal structure of the control group appeared normal under a light microscope. Pathological changes in the ileal tissue were observed in the other groups to varying extents. The ANP and 2:1 groups exhibited severe submucosal hyperemia, edema, and hemorrhage, and even mucosal necrosis and abruption. However, the 1:2 and PAAF groups exhibited mucosal injury and submucosal inflammatory cell infiltration (Fig. 1, *D* and *F*). In addition, we examined the level of inflammation-related markers (TNF-α and IL-6) in ileum tissues. The results revealed that inflammation was the most severe in the ANP and 2:1 groups and least severe in the PAAF group (Fig. 1, *G* and *H*).

Taken together, when compared with the control group, the modified Aho model and the ANP-IR models exhibited similar pathological changes, but with varying degrees of severity. Among the ANP-IR model groups, the pathological changes in the 2:1 group of rats were most similar to those in the ANP group, whereas those in the PAAF group were least pronounced.

### Intestinal Barrier Function and Motility Were Decreased in Different Groups

**Figure 2.**
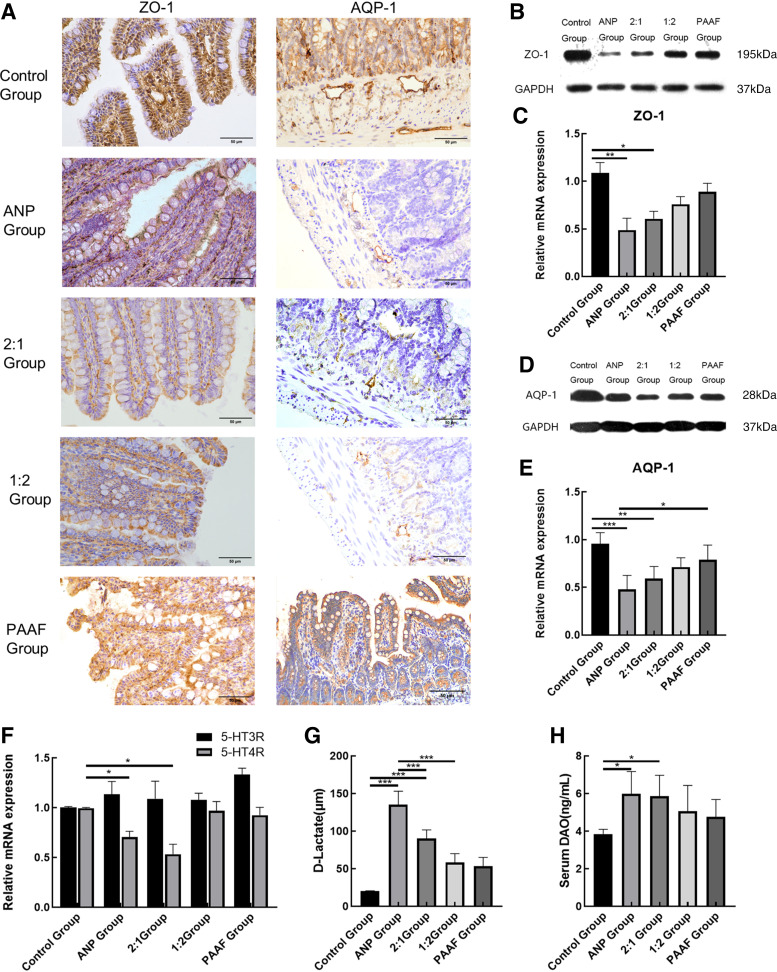
Intestinal barrier function and motility were decreased in different groups. *A*: zonula occludens 1 (ZO-1) and anti-aquaporin-1 (AQP-1) expression were detected through immunohistochemistry (scale bar = 50 μm). ZO-1 and AQP-1 proteins are stained brown. *B* and *D*: Western blotting results showing the expression of ZO-1 and AQP-1. *C* and *E:* RT-qPCR results for the expression of ZO-1 and AQP-1. *F*: RT-qPCR results for the expression of 5-HT3R and 5-HT4R. *G*: serum d-Lactate (d-Lac). *H*: serum DAO. Data are expressed as the means ± SD, *n* = 8 rats in each group, *P < 0.05, **P < 0.01, ***P < 0.001.

We examined changes in ZO-1 and AQP-1 protein levels in rat ileal tissues through immunohistochemistry and Western blot. The results revealed that the ZO-1 and AQP-1 levels were decreased in the ANP group and the ANP-IR model group, and the differences were statistically significant (Fig. 2, *A*, *B*, and *D*). Subsequently, we performed RT-qPCR to determine the relative mRNA expression levels of ZO-1, AQP-1, 5-HT3R, and 5-HT4R in rat ileal tissues. The results demonstrated that the relative expression of ZO-1 mRNA was significantly lower in the ANP and 2:1 groups than in the control group, and the difference was statistically significant (*P* < 0. 05; Fig. 2*C*). Furthermore, the ANP, 2:1, and 1:2 groups exhibited significantly lower expression of AQP-1 mRNA (*P* < 0.05) than the control group (Fig. 2*E*). The expression of 5-HT4R mRNA was significantly lower in the ANP and 2:1 groups than in the control group (*P* < 0.05). However, the expression of 5-HT3R mRNA did not significantly differ between the ANP and 2:1 groups and the control group (Fig. 2*F*).

To examine intestinal barrier function, we determined the serum d-Lac and DAO levels in each group through ELISA. The results revealed that the serum d-Lac and DAO levels were significantly increased compared with those in the control group. Among them, the most significant increase was noted in the ANP group. Among the ANP-IR model groups, the most significant increase was observed in the 2:1 group (Fig. 2, *G* and *H*). The increase in the serum d-Lac and DAO levels indicated that intestinal permeability was altered.

### Levels of Intestinal Apoptosis Increased in Different Groups

**Figure 3.**
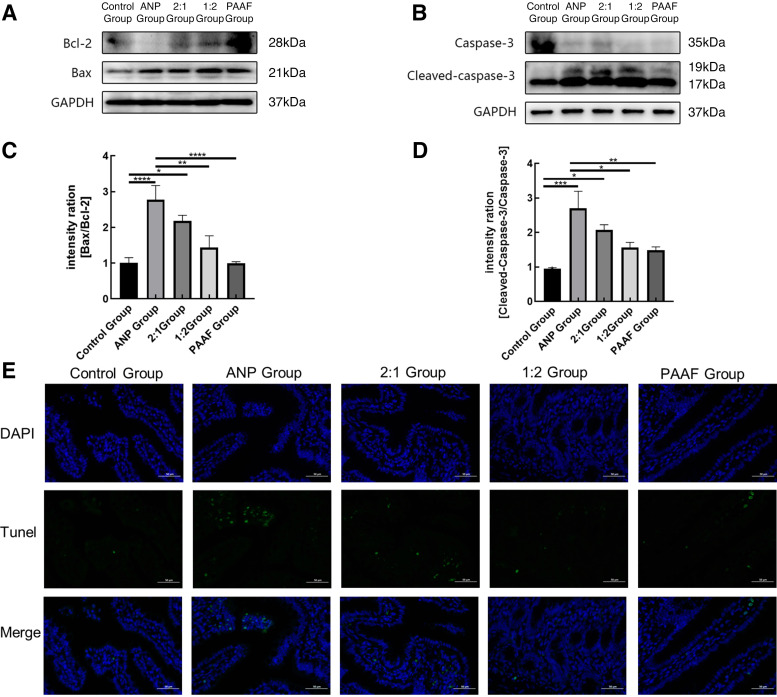
Levels of intestinal apoptosis increased in different groups. *A* and *B*: Western blotting results showing the expression of Bax, Bcl-2, caspase-3, and cleaved-caspase-3. *C* and *D*: the ratios Bax/Bcl-2 and cleaved-caspase-3/caspase-3, and the results are expressed in the histogram. *E*: images depicting TUNEL staining were captured from each of the experimental groups, with a scale bar measuring 50 μm. The green color represents TUNEL-positive nuclei, whereas DAPI staining is represented by blue. Data are expressed as the means ± SD, *n* = 8 rats in each group, *P < 0.05, **P < 0.01, ***P < 0.001, ****P < 0.0001.

Several studies have demonstrated that ANP-associated intestinal injury involves intestinal epithelial necrosis and apoptosis (20–22). Therefore, we evaluated the ANP-IR model by detecting apoptosis-related indices in the intestinal epithelial cells of rats in each group. The expression of apoptosis-related indicators in rat ileal tissues was determined through Western blotting. The apoptosis level was determined based on the ratios of cleaved-caspase-3/caspase-3 and Bax/Bcl-2. The ratios, cleaved-caspase-3/caspase-3 and Bax/Bcl-2, were significantly higher in the ANP and ANP-IR model groups than in the control group (Fig. 3, *A*–*D*). In addition, the number of TUNEL-positive cells was significantly higher in the ANP and the ANP-IR model groups than in the control group (Fig. 3*E*), suggesting the occurrence of varying levels of apoptosis in the ileal tissues.

### Analysis of Cell Experiment Results

**Figure 4.**
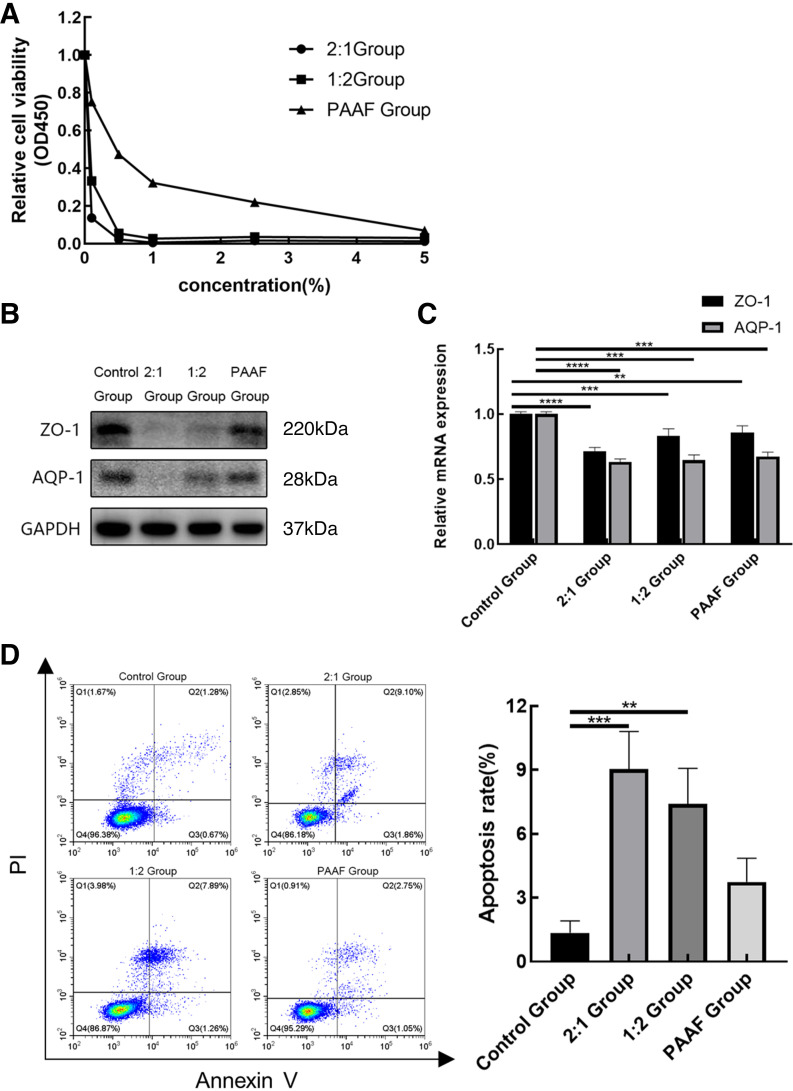
Analysis of cell experiment results. *A*: normal epithelial crypt cells (IEC-6) were treated with a mixture of pancreatitis-associated ascites fluid (PAAF) and necrotic pancreatic tissues in different proportions for 24 h, and cell viability was assayed. *B* and *C*: Western blotting and qPCR results showing the expression of zonula occludens 1 (ZO-1) and anti-aquaporin-1 (AQP-1). *D*: the apoptosis level was determined through flow cytometry. The flow cytometric analysis findings are displayed in the form of a histogram, apoptosis rate is calculated by adding the areas of Q2 and Q3. Data are expressed as the means ± SD, **P < 0.01, ***P < 0.001, ****P < 0.0001. PI, propidium iodide.

Liu et al. (11, 12) simulated the peritoneal inflammation environment by adding peritoneal lavage fluid to the culture medium to investigate the effect of peritoneal macrophage polarization on the treatment of ANP through peritoneal puncture and drainage. In this study, we simulated the accumulation of fluid and necrotic material in rat small intestinal cells (IEC-6) by adding a mixture of PAAF and necrotic pancreatic tissues in varying proportions to the culture medium (0%, 0.1%, 0.5%, 1%, 2.5%, and 5%). After a 24-h treatment period, we examined the survival of IEC-6 cells by using the CCK-8 assay. Our results revealed a gradual decline in the cell survival rates as the proportion of necrotic pancreatic tissues increased, with the 2:1 group demonstrating the most pronounced decrease (Fig. 4*A*). We chose the 0.5% concentration with more significant differences between groups for the subsequent experiments.

We harvested IEC-6 cells treated with a 0.5% mixture for 24 h and examined the levels of ZO-1 and AQP-1 protein and mRNA through Western blot and RT-PCR (Fig. 4, *B* and *C*). The trends observed in cell experiments were similar to those observed in previous animal experiments.

Furthermore, apoptosis was detected in the aforementioned four groups of cells through Annexin V/propidium iodide staining (Fig. 4*D*). These apoptosis levels are consistent with the results of previous animal experiments. The 2:1 group had the highest apoptosis rate, whereas the 1:2 group had the lowest apoptosis rate compared with the control group.

These results suggest that in a simulated model of intestinal damage associated with the accumulation of necrotic material in ANP, the mixture of PAAF and necrotic pancreatic tissue significantly affected IEC-6 cells, including reduced viability of cells and downregulation of protein and mRNA levels associated with intestinal barrier function. In addition, the observed level of apoptosis in the cell assay corroborated the results of animal studies. These findings provide crucial insights into the mechanisms underlying pancreatitis-associated intestinal injury.

### ANP-Associated Intestinal Injury May Be Caused by Decreased Yap Expression

We examined the possible mechanisms for ANP-associated intestinal injury. The Hippo pathway is critical in regulating intestinal homeostasis and regeneration and in maintaining the structural and functional integrity of the epithelial barrier (23–25).

**Figure 5.**
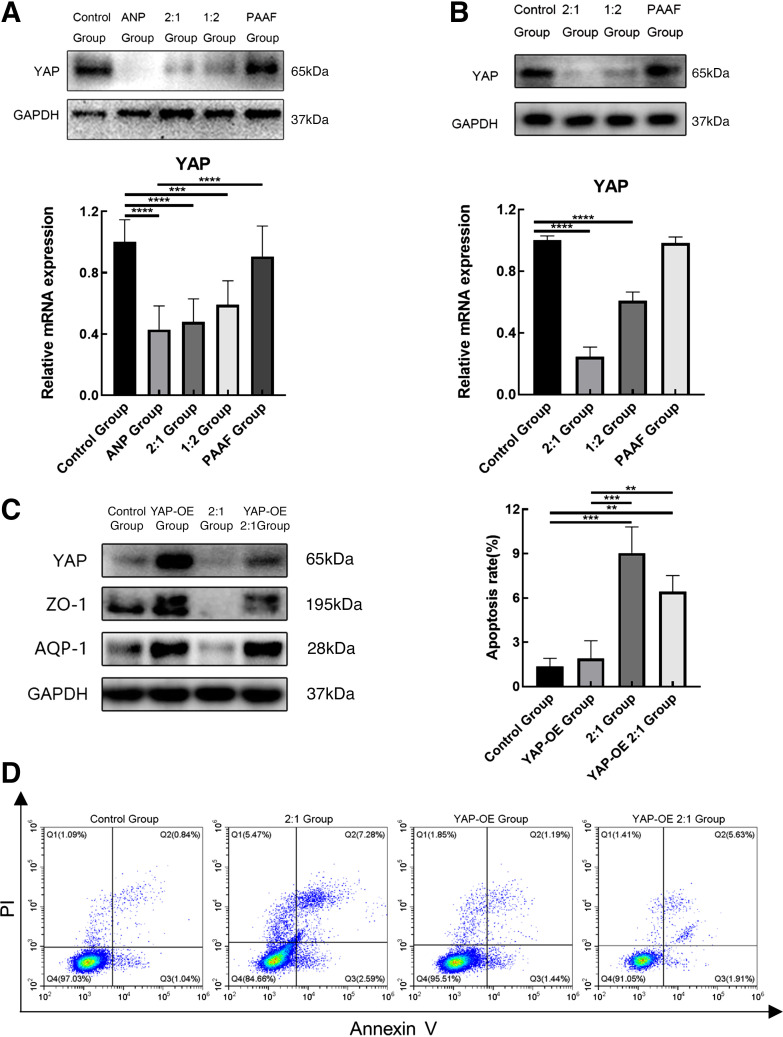
Acute necrotizing pancreatitis (ANP)-associated intestinal injury may be caused by decreased yes-associated protein (YAP) expression. *A*: the levels of YAP protein and mRNA expression in ileal tissues of the rat model were analyzed in each group using Western blot and qPCR. *B:* the levels of YAP protein and mRNA expression in epithelial crypt cells (IEC-6) were analyzed using Western blot and qPCR. *C*: Western blotting showing the expression of YAP, zonula occludens 1 (ZO-1), and anti-aquaporin-1 (AQP-1). *D*: the levels of apoptosis were determined through flow cytometry analysis. The flow cytometric analysis findings are displayed in the form of a histogram, apoptosis rate is calculated by adding the areas of Q2 and Q3. Data are expressed as the means ± SD, **P < 0.01, ***P < 0.001, ****P < 0.0001. PI, propidium iodide.

We detected the expression of YAP protein in the intestinal tissues of rats in each group and found that the YAP protein level was reduced to different degrees in each group (Fig. 5*A*). Next, we examined YAP expression in IEC-6 cells treated with 1:2 and 2:1 mixture of PAAF, and necrotic pancreatic tissues for 24 h and observed reductions in the YAP protein level (Fig. 5*B*). YAP gene was overexpressed in IEC-6 cells, and the cells were then treated with a 2:1 mixture. We determined that compared with normal IEC-6 cells, IEC-6 cells overexpressing YAP protein exhibited less reductions of ZO-1 and AQP-1 protein levels (Fig. 5*C*). Furthermore, we analyzed the apoptosis of the aforementioned four cell groups using Annexin V/propidium iodide staining (Fig. 5*D*). The results indicate that the level of apoptosis in IEC-6 cells overexpressing YAP protein and treated with the 2:1 mixture was lower than that of normal IEC-6 cells treated with the same mixture. Overexpression of YAP reduces the apoptotic effects of necrotic pancreatic tissue and ascites on IEC-6 cells. These findings indicate that the decreased expression of YAP protein may be one of the pathways leading to the apoptosis of small intestinal cells in ANP-associated intestinal injury.

### Relationship between the Severity of AP and Clinical Features

In a prospective randomized trial of patients with moderate-to-severe AP with intestinal dysfunction, Wang et al. used a series of objective measures to examine intestinal functions, including abdominal pain, distention, nausea, and tolerance to enteral nutrition. In addition, the length of hospital stay, mortality rate, tolerance rate of total enteral nutrition on *day 7* after admission, and days to recovery for total enteral nutrition were examined as the basis for a comprehensive evaluation of intestinal function recovery (26–28).

We retrospectively collected data from 1,116 patients with AP who were treated at the Pancreatitis Diagnosis and Treatment Center of the First Affiliated Hospital of Wenzhou Medical University from 2017 to 2020. After further screening based on the inclusion and exclusion criteria, we included 103 patients in this study (Supplemental Fig. S2). Then, we collected their demographic information and clinical characteristics and divided them into five groups. The baseline characteristics were evenly distributed among the five groups (Supplemental Table S2). No significant difference in the total enteral nutrition tolerance rate on *day 7* was noted among the five groups (8.3% vs. 42.9% vs. 30% vs. 45.5% vs. 9.1%, *P* = 0.06). Significant differences were noted among the groups in the total enteral nutrition recovery days (*P* = 0.001), incidence of organ failure (8.3% vs. 0% vs. 2.5% vs. 24.2% vs. 36.4%, *P* = 0.009), and length of hospital stay (*P* = 0.017). Furthermore, no significant differences among the five groups were observed in the incidence of any infectious complication (91.7% vs. 57.1% vs. 77.5% vs. 87.9% vs. 90.9%, *P* = 0.221) and the rate of minimally invasive surgical intervention (16.7% vs. 14.3% vs. 20% vs. 30.3% vs. 27.3%, *P* = 0.761; Table 1).

**Table 1.** Comparison of intestinal function and clinical outcomes between the five groups

Endpoint	A Group (*n* = 12)	B Group (*n* = 7)	C Group (*n* = 40)	D Group (*n* = 33)	E Group (*n* = 11)	*P* Value
*Intestinal function*						
Tolerance rate of complete enteral nutrition on the seventh day of admission [*n* (%)]	1 (8.3)	3 (42.9)	12 (30)	15 (45.5)	1 (9.1)	0.06
The days of recovery of complete enteral nutrition	0 (0, 27)	13 (0, 34)	15 (0, 52)	18 (0, 122)	28 (13, 40)	0.001
The days of SGD falling to 0	12.5 (5, 20)	9 (3, 14)	10 (0, 36)	9 (0, 107)	14 (5, 34)	0.232
*Inflammatory index*						
Any infectious complications [*n* (%)]	11 (91.7)	4 (57.1)	31 (77.5)	29 (87.9)	10 (90.9)	0.221
Bacteraemia [*n* (%)]	0 (0)	1 (14.3)	4 (10)	7 (21.2)	4 (36.4)	
Pneumonia [*n* (%)]	11 (91.7)	4 (57.1)	30 (75)	28 (84.8)	9 (81.8)	
Urinary tract infection [*n* (%)]	0 (0)	1 (14.3)	1 (2.5)	2 (6.1)	0 (0)	
CRP	69.5 (4.65, 289)	31.4 (9, 90)	90 (3.11, 275)	90 (3.23, 307)	90 (7.3, 401)	0.19
D-Dimer	2.28 (0.56, 14.12)	2.11 (0.22, 13.28)	2.55 (0.49, 20)	3.89 (0.45, 20)	4.1 (1.15, 11.09)	0.112
*Severity*						
Any organ failure [*n* (%)]	1 (8.3)	0 (0)	1 (2.5)	8 (24.2)	4 (36.4)	0.009
Respiratory failure [*n* (%)]	0 (0)	0 (0)	1 (2.5)	7 (21.2)	2 (18.2)	
Renal failure [*n* (%)]	1 (8.3)	0 (0)	0 (0)	3 (9.1)	2 (18.2)	
Circulatory failure [*n* (%)]	0 (0)	0 (0)	0 (0)	3 (9.1)	1 (9.1)	
Intervention of surgery [*n* (%)]	2 (16.7)	1 (14.3)	8 (20)	10 (30.3)	3 (27.3)	0.761
Hospital stay, days	16.5 (13, 41)	14 (11, 49)	21 (11, 57)	25 (12, 125)	31 (16, 107)	0.017

Data are given as the *n* (%) where *n* represents number of rats, mean (standard deviation) or median (range). CRP, creatine phosphate;

SGD, score of gut dysfunction.

## DISCUSSION

The modified Aho ANP model simulates clinical biliary reflux pancreatitis through retrograde injection via the pancreatic bile duct. However, there are three limitations in studying ANP-associated intestinal injury using the modified Aho ANP model: *1*) poor stability: the modified Aho ANP model is not designed specifically to study ANP-associated intestinal injury. Therefore, the model may not be able to induce intestinal injury consistently, which could result in a waste of resources during the study. *2*) Second-order injury: Although external pancreatic organ injuries can be observed after modeling, these injuries fall under the category of “second-order injury” after pancreatitis injury, making it challenging to control the extent of secondary organ damage. *3*) Anatomical differences: The human pancreas is a retroperitoneal organ, and local necrotic tissues can easily spread along the retroperitoneum to other areas. In contrast, the rat pancreas is an intraperitoneal organ. *4*) Intestinal injury bias: To establish a rat ANP model through the retrograde injection of sodium taurocholate into the bile-pancreatic duct, it is necessary to simulate intestinal injury. However, if this model is used to investigate ANP-associated intestinal injury, it becomes difficult to exclude the possibility of intestinal injury resulting from the pinhole and pouch suture technique used during model establishment (29).

Clinical evidence has firmly established the close relationship between peripancreatic acute fluid accumulation, acute necrotic material accumulation, and the severity of ANP. Adequate drainage of the accumulated peripancreatic acute fluid and acute necrotic materials can alleviate ANP (30). In addition, the 2012 International Consensus on the Classification of AP classifies gastrointestinal injury as a local complication of ANP, further strengthening the connection between ANP events such as acute fluid accumulation and acute necrotic material accumulation with gastrointestinal injury (1). Sugimoto et al. (31) reported that PAAF contains numerous toxic substances, such as trypsin, cytokines, and endotoxins, resulting in various direct or indirect injuries. Ranson et al. (32) demonstrated that peritoneal lavage could ameliorate the clinical symptoms of ANP and reduce the early mortality of ANP, confirming the detrimental effects of PAAF. Fujita et al. (33) successfully induced AP lung injury in rats through the intraperitoneal injection of PAAF. Based on these research findings, we designed an ANP-IR model to simulate intestinal injuries caused by acute fluid and necrotic matter accumulation by injecting PAAF in combination with varying proportions of necrotic pancreatic tissues into the triangular area formed by the left renal artery and ureter in rats.

Our experimental results demonstrated that both the ANP-IR model and the modified Aho ANP model effectively induced intestinal injury in rats. This injury was predominantly characterized by varying degrees of submucosal hyperemia, edema, and hemorrhage in the ileal tissue, and even mucosal necrosis and sloughing in some cases. In the ANP-IR model, the severity of systemic inflammation differed depending on the proportion of ascites and necrotic pancreatic tissue injected into the homogenate, with the necrotic pancreatic in the homogenate potentially being the primary cause of pancreatitis-associated intestinal function injury. Subsequently, we confirmed that ZO-1 protein and mRNA levels were decreased in the ileal tissues of rats in each group. ZO-1 protein is involved in the formation and maintenance of the structure of tight junctions between cells, forming a continuous and complete barrier on the cell surface by interacting with other tight junctions (34–36). The decreased expression of ZO-1 protein, which hinders the free passage of substances and the expansion of intercellular gaps, indicated varying degrees of damage to the intestinal barrier function in all model groups. AQP-1 functions as a water channel in small intestinal epithelial cells and increases water absorption capacity by promoting the rapid transport of water molecules (37–39). Feng et al. (40) found the downregulation of AQP-1 expression in the pancreas, lung, and intestinal tissues of ANP rats. In addition, Lu et al. (37) observed a decrease in AQP-1 expression in the small intestinal tissues of ANP-induced rats. They believed that the decrease in AQP-1 expression caused edema in the small intestine as well as damaged the small intestinal microcirculation and small intestine in ANP rats. Our study further corroborated AQP-1 exhibited reduced expression levels. This reduction impaired the small intestine’s ability to absorb water, leading to water retention within the intestine and subsequently contributing to ANP-associated intestinal injury, characterized by intestinal edema. In addition, the upregulation of 5-HT3R expression may be attributed to the activation of 5-HT3R, which led to increased intestinal smooth muscle contraction and sensory neurotransmission during the early stages of acute intestinal injury, thereby causing increased pain and intestinal peristalsis. The decrease in 5-HT4R expression may be linked to the fact that 5-HT4R promotes intestinal peristalsis and gastrointestinal secretion. Decreased gastrointestinal activity may contribute to wound healing and prevent further injury.

**Figure 6.**
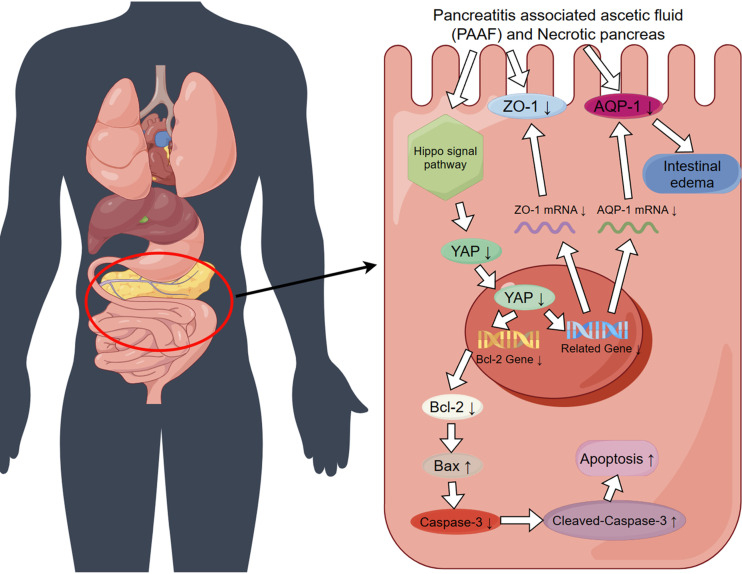
Schematic diagram of intestinal injury induced by a mixture of pancreatitis-associated ascites fluid (PAAF) and necrotic pancreatic tissues.

We examined apoptosis-related indicators in the ileal tissues of rats in each group and observed varying levels of apoptosis among the different groups, which was also verified at the cellular level. The Hippo pathway is crucial for the regulation of intestinal homeostasis and regeneration and maintenance of the structural and functional integrity of the epithelial barrier (23–25). Therefore, we measured the YAP protein level in the ileal tissues of rats in each group and noted a decrease in its level to varying degrees across the groups. Combined with our previous findings, we speculated that intestinal injury associated with ANP may partly result from the activation of the Hippo pathway, leading to the phosphorylation of YAP in the cytoplasm. A portion of phosphorylated YAP is subsequently degraded, whereas the rest remains in the cytoplasm as phosphorylated YAP. As YAP levels decrease in the nucleus, the ability of YAP to promote cell proliferation and inhibit apoptosis decreases, resulting in elevated levels of apoptosis-related markers and enhanced apoptosis. In addition, some intestinal damage may be related to the decreased expression of ZO-1. Kim et al. (41) found that in E-cadherin-restored AGS gastric cancer cells, ZO-1 and YAP, exerted a mutual regulatory effect on YAP-mediated cell migration. In other words, the decreased expression of YAP or ZO-1 reduces the expression of the other protein (41, 42). Therefore, in ANP-associated intestinal injury, the decreased expression of ZO-1 in the intestinal epithelial cells leads to the decreased expression of YAP, resulting in the decreased entry of YAP into the nucleus and thus weakening the effect of YAP on promoting cell proliferation and inhibiting cell apoptosis. In addition, apoptosis-related indices were increased and apoptosis-related effects were enhanced. To verify our hypothesis, we overexpressed the *YAP* gene in IEC-6 cells. The experimental results revealed that when the expression of YAP protein was increased, the apoptosis-related indices decreased significantly. However, the expression level of ZO-1 did not change significantly. Based on these experimental results, we believe that when ANP occurs, the activation of the Hippo pathway in the ileum may lead to the apoptosis of intestinal cells (Fig. 6).

To determine the relationship between the severity degree of ANP and various prognostic indicators, we collected data on the clinical characteristics of 103 patients diagnosed as having ANP based on the modified Balthazar CT score. The results revealed that patients with a greater ratio of pancreatic necrosis to abdominal effusion required more days for complete recovery of enteral nutrition, indicating that abdominal effusion and pancreatic necrosis may be key factors affecting the recovery of intestinal function. Combined with previous findings, we believe that these changes may compromise intestinal function and intestinal barrier integrity, and the accumulation of pancreatic necrosis may be the primary cause of intestinal injury. In addition, we found that the greater the ratio of pancreatic necrosis to abdominal fluid, the greater the likelihood of organ failure. This finding suggests that abdominal fluid accumulation and pancreatic necrosis caused by ANP not only affect intestinal function but also cause damage to other organs to some extent, potentially leading to the occurrence of multiple organ failure. This finding has crucial implications for clinicians in assessing the severity of AP. In conclusion, we demonstrated the relationship between the presence or absence of abdominal fluid and pancreatic necrosis in patients with ANP with intestinal injury and multiple organ failure. This finding enhances our understanding of the pathophysiological processes of AP, providing crucial insights for the development of more effective treatment.

Overall, this modeling process, characterized by its simplicity, repeatability, singular damage factor, and controlled damage degree, provides a more ideal animal experimental model for investigating the mechanisms of ANP-associated intestinal injury. However, due to individual differences among rats, each modified Aho model rat may exhibit instability. Furthermore, the PAAF and necrotic pancreas of each modified Aho model rat have limitations, resulting in a restricted number of ANP-IR model rats that can be produced using the same modified Aho model rat. Therefore, differences may exist between the ANP-IR model rats produced using PAAF and necrotic pancreas from different individuals. However, we can minimize differences by standardizing and ensuring uniformity in the modeling process of the modified Aho model rats. This will reduce the variation of the ANP-IR models produced by different individual rats. It is important to use rats with the same genetic background to minimize individual differences and the possibility of rejection.

### Perspectives and Significance

This study verified the feasibility and advantages of the ANP-IR model by comparing it with the modified Aho ANP rat model. Furthermore, we used the ANP-IR model to elucidate the mechanisms underlying ANP-associated intestinal injury. In addition, at the clinical level, we confirmed the detrimental effects of PAAF and pancreatic necrotic tissues on the intestine of patients with ANP. Our study introduced an ANP-associated intestinal injury rat model characterized by a single injury factor, a controllable degree of injury, and low and manageable mortality rates. This mode is more in line with human anatomy. Our results indicated that the occurrence of ANP intestinal injury may be associated with the decreased expression of YAP in the Hippo pathway. Targeting this pathway presents a potential therapeutic approach for the treatment of ANP-associated intestinal injury. In addition, it provides a theoretical basis for patients with ANP, emphasizing the importance of the timely removal of accumulated peritoneal necrotic materials to promote the recovery of intestinal function and enhance therapeutic outcomes.

## DATA AVAILABILITY

All data generated and analyzed during this study are included in this article and supplemental file.

## SUPPLEMENTAL DATA

10.6084/m9.figshare.25664019 Supplemental Figs. S1 and S2 and Supplemental Tables S1 and S2: https://doi.org/10.6084/m9.figshare.25664019.

## GRANTS

This study was supported by the Wenzhou Municipal Science and Technology Plan Project (Y20210184) and the Natural Science Foundation of Zhejiang Province (LQ24H070008).

## DISCLOSURES

No conflicts of interest, financial or otherwise, are declared by the authors.

## AUTHOR CONTRIBUTIONS

H.J., W.X., Z.Y., and N.L. conceived and designed research; H.J. and Q.C. performed experiments; H.J. and Q.C. analyzed data; H.J., W.X., and N.L. interpreted results of experiments; H.J. prepared figures; H.J., W.X., Y.L., Z.Y., and N.L. edited and revised manuscript; H.J. and N.L. approved final version of manuscript.
